# Perceptual learning with tactile stimuli in rodents: Shaping the somatosensory system

**DOI:** 10.3758/s13420-017-0269-y

**Published:** 2017-04-21

**Authors:** Nicole Pacchiarini, Kevin Fox, R. C. Honey

**Affiliations:** Cardiff University, Cardiff, UK

**Keywords:** Associative learning, Discrimination, Generalization, Habituation

## Abstract

The animal kingdom contains species with a wide variety of sensory systems that have been selected to function in different environmental niches, but that are also subject to modification by experience during an organism’s lifetime. The modification of such systems by experience is often called *perceptual learning*. In rodents, the classic example of perceptual learning is the observation that simple preexposure to two visual stimuli facilitates a subsequent (reinforced) discrimination between them. However, until recently very little behavioral research had investigated perceptual learning with tactile stimuli in rodents, in marked contrast to the wealth of information about plasticity in the rodent somatosensory system. Here we present a selective review of behavioral analyses of perceptual learning with tactile stimuli, alongside evidence concerning the potential bases of such effects within the somatosensory system.

Psychological theories of learning and memory in nonhuman animals have been shaped by the investigation of rodents confronted with more-or-less complex experimental events constructed from a limited palate of stimuli. The associative models of learning and memory that have developed on the basis of such investigations are correspondingly frugal: They seek to explain learned changes in behavior—that is, learning in general—through the operation of a relatively small set of primitive processes that are held to be common across a broad range of sensory environments (e.g., Pavlov, 1941). Very briefly, stimuli are assumed to activate sets of representational elements, which become linked when they are active at the same time. The resulting association allows future activation of one set of elements to reactivate the now-absent set, with associative change being the sole form of plasticity within at least some models of associative learning (e.g., Wagner, 1981). Although the critical research on the nature of associative processes in animals has employed a limited set of stimuli (e.g., auditory and visual stimuli), it is assumed that the processes identified have broad explanatory power. In some cases this assumption has been supported directly (e.g., Koskal, Domjan, & Weisman, 1994; Rodrigo, Chamizo, McLaren, & Mackintosh, 1997), whereas in other cases the need to make use of additional stimulus dimensions (e.g., tactile stimuli) has created de facto support for its validity (e.g., Allman, Ward-Robinson, & Honey, 2004; Lawrence, 1949). However, there has often been little attempt to understand which aspects of the physical properties of the chosen stimulus dimensions are important. This fact has been compounded by a lack of contact with researchers whose main interests are in the requisite transduction of sensory signals and the plasticity of sensory systems of the brain. One area in which this lack of integration is especially perplexing is in the study of perceptual learning.

Gibson (1963, p. 29) defined perceptual learning as “Any relatively permanent and consistent change in the perception of a stimulus array, following practice or experience with this array.” Here, the term *perceptual learning* is used to refer to the observation that simple exposure to two (similar) stimuli increases the ease with which they are later discriminated from one another. For example, in the classic demonstration of perceptual learning, rats were raised from birth in white cages with black geometric forms (e.g., a black circle and a black triangle) suspended against their walls (Gibson & Walk, 1956). When the rats were 90 days old, they received a discrimination in a Lashley jumping stand in which approaching one form (e.g., the circle) enabled access to food and approaching the other (e.g., the triangle) did not. The rats that had received extensive preexposure to the two stimuli acquired the discrimination more rapidly than those who encountered the stimuli for the first time during discrimination training. This observation has been replicated in adult rats (Channell & Hall, 1981) and has been analyzed using a range of nonhuman species (e.g., rats, pigeons, and chicks) and stimuli (e.g., flavors or visual stimuli; for recent reviews, see Mitchell & Hall, 2014; Montuori & Honey, 2014). The case under consideration here involves the use of tactile stimuli in rodents. We begin by reporting the results of a recent behavioral analysis of the effects of experience with tactile stimuli on later discrimination learning, and proceed by examining the potential locus of this experience-dependent behavioral plasticity within the rodent somatosensory system, where we are guided by a burgeoning literature (see Fox, 2008).

## Perceptual learning with tactile stimuli

James (1890) noted that professional commodity traders could “recognize, by feeling the flour in a barrel, whether the wheat was grown in Iowa or Tennessee” (see also Gibson, 1967). Such feats are certainly impressive, as well as consistent with the idea that experience shapes the perception of tactile stimuli, and abundant evidence has now confirmed the fact that difficult tactile discriminations are improved by experience in humans (e.g., Sathian & Zangaladze, 1997; see also Rodríguez & Angulo, 2014). However, until recently the same could not be said of evidence from nonhuman animals, in which studies of perceptual learning have used either flavors (e.g., Honey & Hall, 1989; Mackintosh, Kaye, & Bennett, 1991; Scahill & Mackintosh, 2004; Symonds & Hall, 1995) or visual stimuli (e.g., Channell & Hall, 1981; Gibson & Walk, 1956; Honey, Bateson, & Horn, 1994). Certainly, some research has made use of floor textures to study perceptual learning with rats (Chamizo & Mackintosh, 1989 ; Trobalon, Chamizo, & Mackintosh, 1992; Trobalon, Sansa, Chamizo, & Mackintosh, 1991). However, in these cases the floor textures were used in conjunction with procedures that were not designed to permit the critical effects of prior experience to be attributed to changes in the processing of the floors. In contrast, Montuori and Honey (2016) recently reported an analysis of perceptual learning with floor textures in which such an attribution was warranted, and in which the perceptual learning effect had a clear origin.

**Fig. 1:**
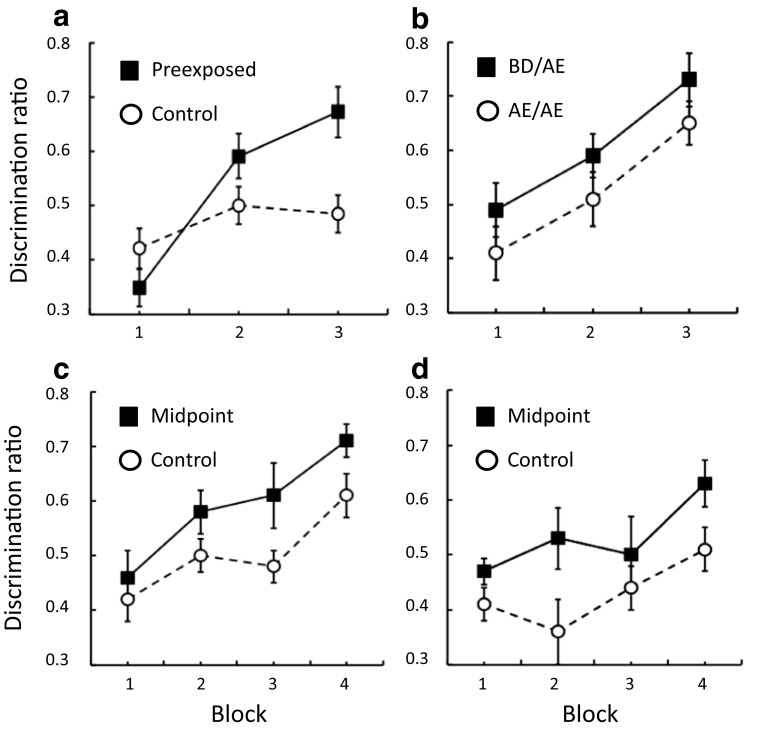
Perceptual learning. Discrimination learning involved stimuli along a tactile dimension created using sandpapers of different grit sizes (A, B, C, D, and E) and was assessed using discrimination ratios: the number of food well entries during the reinforced tactile stimulus (e.g., A) divided by the total number of entries during the reinforced and nonreinforced (e.g., E) tactile stimuli (A+E). Panel A shows that discrimination learning proceeded more rapidly in rats preexposed to A and E than in a control group exposed only to the apparatus. Panel B shows that the discrimination between A and E proceeded more rapidly in rats preexposed to B and D (group BD/AE) than in a group preexposed to A and E (group AE/AE; where the letters before / denote the preexposed stimuli and the letters after / denote the stimuli presented during discrimination training). Panels C and D show that preexposure to a midpoint (C) between the to-be-discriminated stimuli facilitated discrimination learning relative to two control groups. From: Perceptual learning with tactile stimuli in rats: Changes in the processing of a dimension by L. M. Montuori and R. C. Honey, 2016, *Journal of Experimental Psychology*: *Animal Learning and Cognition*, *42*, pp. 283–286. Copyright 2016 by the American Psychological Association. Adapted with permission.

In the first study, on each of four days rats were placed in operant chambers for four 3-min sessions that were separated by 1 min. For the control group the floor was sheet metal, and for the preexposed group this floor was covered with one of two sandpapers (p40 or p100), which correspond to grit sizes of 425 and 162 *μ*m, respectively. These different floors were presented in an alternating sequence. Rats in both groups then received discrimination training in which placement on one texture (A) was paired with food, whereas placement on the other (E) was not; and we measured rats visits to the food well during the periods at the start of each session in which no food was delivered. Inspection of Fig. 1a shows that the rats that had received prior exposure to the two floors learned the discrimination more rapidly than those for whom the floors were novel at the outset of discrimination training—an instance of a perceptual-learning effect involving tactile stimuli.

What is the origin of this perceptual-learning effect? A number of possibilities spring to mind, including the potential contribution of other senses (e.g., visual), which can be excluded on the basis of results that will soon be presented (e.g., by conducting experimental procedures in the dark). However, perhaps the simplest theoretical analysis involves first assuming that the two floors (A and E) activate sets of representational elements, some of which are activated by both A and E (call them *x*), and others that are uniquely activated by A or E (*a* and *e*, respectively; see Atkinson & Estes, 1963). We can speculate that these elements are activated by a rat’s whisker system interacting with the floors, but they might be activated in other ways, too. In any case, armed with this assumption about the nominal elements activated by A and E, it becomes evident that rats preexposed to both stimuli will have encountered *x* on twice as many occasions as either *a* or *e*: The common elements (*x*) are presented on trials with both A and E, and the unique elements (*a* and *e*) are presented only on trials with A or E. This fact could have a number of repercussions, but if it is also assumed that the processing of a given set of elements (*a*, *e*, or *x*) undergoes a reduction in processing on each occasion that it is activated (Lubow, 1973), then *x* will become less well processed than either *a* or *e* as a result of preexposure to A and E. This form of analysis has been entertained on several occasions (see Honey & Hall, 1989; McLaren, Kaye, & Mackintosh, 1989) and need not be aligned to any specific account of the critical reduction in processing (e.g., McLaren & Mackintosh, 2002; Pearce & Hall, 1980; Wagner, 1981). The essence of the analysis is that it is “as if” repeated exposure to AX and EX results in them becoming functionally equivalent to Ax and Ex, where the relative sizes of the letters correspond to the processing that they receive. This state of affairs would mean that discrimination learning would proceed more rapidly after preexposure, because the source of generalization between the to-be-discriminated stimuli (i.e., *x*) has been rendered less effective than the components that discriminate between the two stimuli (i.e., *a* and *e*).

The analysis developed in the immediately preceding paragraph is unlikely to be the sole basis of perceptual learning (seeMcLaren et al., 1989; see also Mundy, Honey, & Dwyer, 2007). However, Montuori and Honey (2016) concluded that it provided an adequate account for the perceptual-learning effect found in their procedure. The basis for this conclusion was a set of experiments that made use of the fact that different grades of sandpaper could be used to create a dimension spanning A, B, C, D, and E, where letters closer together in the alphabet represent sandpapers that have more similar grit sizes. This description of the dimension received support from the simple observation that the reinforced discrimination between A and E proceeded more rapidly than that between B and D. The ensuing experimental analysis began with a simple second study. One group of rats again received preexposure to A and E (P60 and P320 sandpapers; 269 and 46.2 *μ*m, respectively), and another were given preexposure to B and D (here, P80 and P150; 201 and 100 *μ*m, respectively). Both groups then received a discrimination in which being placed on floor A was paired with food and being placed on floor E was not. This discrimination was acquired more rapidly after preexposure to B and D than after preexposure to A and E (see Fig. 1b). In some respects this might come across as a counterintuitive finding, but it is not unprecedented. A similar effect has been observed in rats exposed to flavors (Scahill & Mackintosh, 2004; see also Sanjuán, Nelson, & Alonso, 2014). Moreover, it is predicted by a simple analysis in terms of changes in the processing of the unique and common elements of the textures presented for discrimination training (i.e., A and E). It is predicted because, although preexposure to A and E will result in a greater reduction in the processing of their shared elements (*x*) than of their unique elements (*a* or *e*), this difference will be exaggerated after preexposure to B and D. After preexposure to B and D, the unique elements of A and E (i.e., *a* and *e*) will not have been presented, but their common elements (*x*) will have undergone a reduction in processing.

Perhaps the most direct way to assess the theoretical analysis developed in the previous paragraphs is to examine the effect of preexposure to C (a “midpoint” along the texture dimension) on the acquisition of a discrimination between A and E. Preexposure to the midpoint should be all that is required to produce a perceptual-learning effect, because it will result in a reduction in processing of the elements shared by A and E (*x*) without affecting their unique elements (*a* and *e*). This prediction was tested in two experiments, one in which rats in the control group were preexposed to a chamber with a sheet metal floor, and one in which they were preexposed to the backside of a sheet of sandpaper. Relative to both control groups, preexposure to the midpoint resulted in more rapid discrimination between A and E (see Fig. 1c and d). That is, perceptual learning involving tactile stimuli does not depend upon the rats receiving preexposure to the unique elements of the to-be-discriminated tactile stimuli; in fact, learning is retarded by exposure to these elements, as the results presented in Fig. 1b show. This conclusion makes the task of understanding tactile perceptual learning somewhat more tractable than that of understanding other instances of this effect.

The analysis that has been developed for perceptual learning involving tactile stimuli rests on the assumption that preexposure to a tactile stimulus results in a reduction in the processing that it receives. But where is the direct evidence to support this assumption with tactile stimuli? There is some evidence that discrimination training can result in changes in the processing of tactile stimuli (e.g., Lawrence, 1949; Oswald et al., 2001). There is also evidence that familiarity with an environment in which rats run to gain food results in increased locomotor speed and changes in how the rats deploy their whiskers (Arkley, Grant, Mitchinson, & Prescott, 2014). However, of direct relevance to the claim that simple exposure can affect such changes (in this case, reductions) in the processing of tactile stimuli are the results of a study that adapted a procedure used to assess visual memory in rodents, variously called *spontaneous object recognition* (Warburton & Brown, 2015) or *novel object recognition* (Ennaceur & Delacour, 1988). Here, we prefer to use the less loaded term *habituation* to describe the stimulus-specific reduction in whisker-based exploration of tactile stimuli in rodents that was observed by Wu, Ioffe, Iverson, Boon, and Dyck (2013).

## Short-term tactile memory

**Fig. 2:**
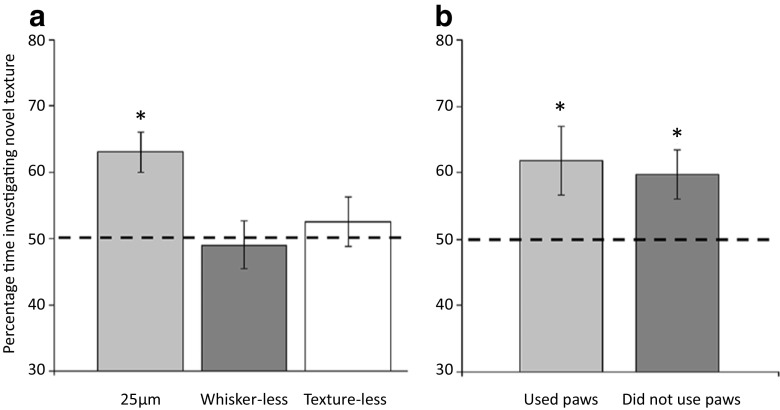
Short-term habituation: Mean percentages of time (±SEM) spent interacting with a novel tactile panel (e.g., 140 *μ*m) relative to a familiar tactile panel (e.g., 115 *μ*m) that had been presented 5 min ago for 5 min. Panel A shows that mice spent more time with a novel than with a familiar stimulus when the two stimuli were separated by 25 *μ*m (i.e., 140-*μ*m and 115-*μ*m sandpapers), and that this effect was absent in whisker-less mice and when the tactile panels were covered in transparent film, and were thus textureless. Panel B shows that the preference to explore the novel panels was evident irrespective of whether mice had interacted with the panels with their paws. From: Novel, whisker-dependent texture discrimination task for mice, by H. P. Wu, J. C. Ioffe, M. M. Iverson, J. M. Boon, and R. H. Dyck, 2013, *Behavioural Brain Research*, *237*, p. 241. Copyright 2013 by Elsevier B.V. Adapted with permission.

The procedure used by Wu et al. (2013) made use of the natural tendencies for rodents (mice) to explore novel stimuli (e.g., with their whiskers) and for this exploration to habituate during a period of exposure (cf. Grion, Akrami, Zuo, Stella, & Diamond, 2016). To assess whether or not this habituation is stimulus-specific, rodents were given a choice between the habituated stimulus and a novel stimulus. When visual stimuli are used, rodents will preferentially explore the novel stimulus and can be said to have recognized the familiar stimulus. Wu et al. placed mice in an open-field arena containing two sandpaper-covered panels (e.g., 140 *μ*m) located in the center of the arena. The mice were given 5 min to explore these panels and were then removed from the arena; 5 min later, they were again placed in the arena, but now with one familiar panel covered with the same sandpaper as during training (e.g., 140 *μ*m) and one panel covered with a novel sandpaper (e.g., 115 *μ*m). The mice spent more time exploring the novel than the familiar tactile panel (see Fig. 2a).

The procedure described above clearly required the mice to have some memory of the tactile stimulus that had been presented 5 min ago. Wu et al. (2013) also provided evidence that this example of stimulus-specific habituation reflected whisker-based interactions with the tactile properties of the stimulus: The effect was not observed in whisker-less mice or if the panels were rendered textureless by being covered with a film that allowed for visual but not tactile interaction (see Fig. 2a), but it was observed irrespective of whether or not the mice had interacted with the stimuli with their paws (see Fig. 2b). These observations converge on the suggestion that the stimulus-specific habituation was a product of whisker-based interactions with the panels. However, the analysis of perceptual learning developed from the perceptual-learning effects reported by Montuori and Honey (2016) requires that such habituation effects be long-term, lasting from one day to the next: Their preexposure stage was separated from the discrimination-learning stage by approximately 24 h. Habituation effects—both short- and long-term—can be explained in a variety of ways (e.g., Groves & Thompson, 1970; Horn & Hill, 1964 ; Konorski, 1967; Sokolov, 1960). However, one associative account of habituation assumes that the behavioral effect reflects the fact that an association forms between the experimental context (in this case, the arena) in which the animal is placed and the stimulus that is presented there. This association renders the presentation of the stimulus unsurprising when it is presented in the context in which preexposure occurred (e.g., Wagner, 1981). Our recent research indicates that the stimulus-specific habituation effect observed by Wu et al. (2013) can be retained for at least 24 h.

## Long-term tactile memory

**Fig. 3:**
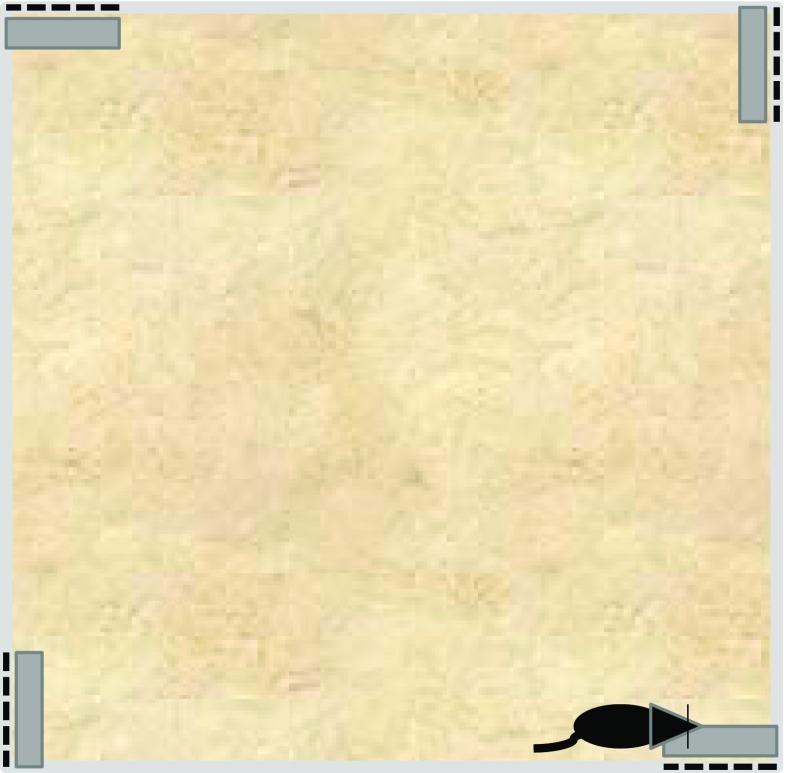
Schematic of the apparatus used to study long-term habituation. The tactile panels are represented by hashed lines, and the arena by the large gray square. During habituation training, the texture gratings on the four panels were the same (e.g., horizontally oriented); during the test, the gratings on two of the panels were the same as during training (e.g., horizontal ), whereas the gratings on the two remaining panels were novel (e.g., vertical ). The amount of time spent in the vicinity of the panels (indicated by the gray rectangles) was measured.

We placed mice in an arena (50 cm^2^) in an experimental room that was illuminated with infrared light, which allowed the behavior of the mice to be captured using a video camera but did not permit the mice to see the black, 3-D-printed tactile panels. The tactile panels were 80 × 80 mm square and 3 mm thick; contained grooves 1.0 mm deep, 0.6 mm wide, and 1.9 mm apart; and could be oriented so that the gratings were either horizontal or vertical. The ability to manufacture textured panels provides a degree of control over the nature of the stimulus that is not possible with commercially available sandpapers. The behavioral recordings were scored using automated software that tracked whether the mice were within a whisker’s distance of the panel (see the small gray rectangles in Fig. 3). On the first day, mice were placed in the arena for two 5-min sessions that were separated by 5 min. The arena contained four identical wall-mounted panels with one grating (e.g., horizontal) that were fixed to the four corners of the arena (see Fig. 3). This way of presenting the panels was chosen to ensure that our mice—who we found tended to explore the perimeter of the arena rather than its center—would encounter the panels on a frequent basis. On the test day, approximately 24 h later, the mice were placed in the arena for 5 min, during which two of the panels were in the same orientation as during training (e.g., horizontal) and two were reoriented (e.g., vertical); all of the panels had been cleaned with ethanol. We assumed that these orientations would be distinguishable to the mice. The question of principal interest was whether the mice would be more likely to interact with the novel gratings than with the preexposed, familiar gratings. We examined the first 30-s epoch during which the mice visited more than one corner (often the first epoch). During this epoch, the mice spent a mean of 65.20% of their time with the novel panel gratings [*SEM* = 5.23; one-sample *t* test against a value of 50%: *t*(19) = 2.91, *p* < .01]. These results replicated and extended those of Wu et al. (2013) and demonstrated, albeit in a different species than had been used by Montuori and Honey (2016), the required longevity of stimulus-specific habituation; an index of the reduction in stimulus processing.

These results show that the exploration of tactile stimuli declines as the result of experience, and provide an empirical foundation for the view that perceptual learning involving tactile stimuli might reflect changes in the distribution of processing between the common and unique elements of two tactile stimuli. The results reported by Wu et al. (2013), together with our extension of that research, suggest that the exploration of tactile stimuli (e.g., through whisking; Carvell & Simons, 1990; Woolsey & Van der Loos, 1970) changes as a product of experience with them (cf. Arkley et al., 2014). The neural bases of these changes are of immediate significance to gaining a more complete understanding of the nature of perceptual learning.

## The processing of tactile stimuli in rodents

**Fig. 4:**
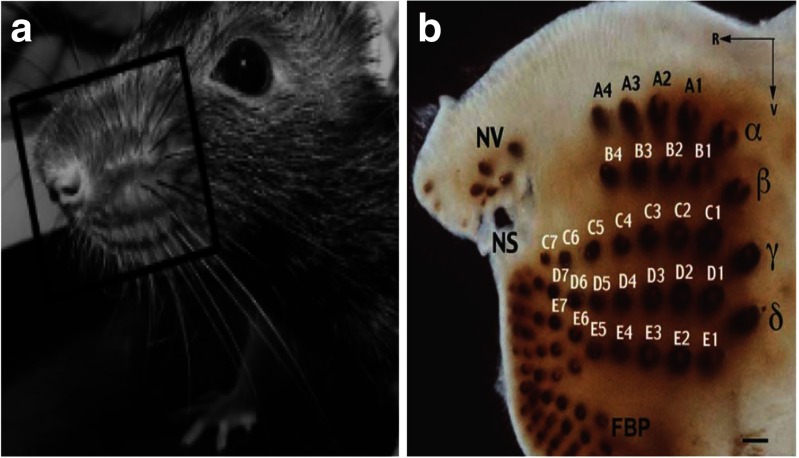
Rodents have two sets of whiskers: macrovibrissae located on the snout, and smaller microvibrissae on the chin and nose. (A) Photograph of a rat head, with mystacial whiskers and pad indicated by the black box. From: The evolution of active vibrissal sensing in mammals: Evidence from vibrissal musculature and function in the marsupial opossum *Monodelphis domestica*, by R. A. Grant, S. Haidarliu, N. J. Kennerley, & T. J. Prescott, 2013, *Journal of Experimental Biology*, *216*, p. 3486. Copyright 2013 by The Company of Biologists Ltd. Adapted with permission. (B) Xylene-cleared section of the macrovibrissal follicles in the rat: *α*–*δ* = the four most caudal vibrissa follicles (“straddlers”); A–E = the five vibrissal rows; FBP = furry buccal pad; NS = nostril; NV = nasal vibrissae; R = rostral; V = ventral; scale bar = 1 mm. From “Muscle architecture in the mystacial pad of the raat, by S. Haidarliu, E. Simony, D. Golomb, & E. Ahissar, 2010, *Anatomical Record*, *293*, p. 1194. Copyright 2010 by Wiley-Liss, Inc. Adapted with permission.

As we have briefly mentioned, rodents explore environments with their facial whiskers (also referred to as *vibrissae*; Vincent, 1912), which are thin, tapered rods (approximately 30 mm/3,000 *μ*m in length) located on each side of their faces that serve as sensors for tactile information (Sofroniew, Cohen, Lee, & Svoboda, 2014). In fact, rats and mice have two sets of whiskers: the macrovibrissae, which are a large matrix of about 25 motile sensors on either side of the snout, and the microvibrissae, which are shorter whiskers around the mouth, chin, and nose of the animal (Deschenes, Moore, & Kleinfeld, 2012). The whiskers are organized in a grid that is made up of five rows (labeled A to E; see Fig. 4; Diamond & Arabzadeh, 2013) and are used to palpate objects through an active process known as “whisking,” involving fast, large-amplitude rhythmic sweeping movements of the macrovibrissae (Carvell & Simons, 1990; Knutsen, Derdikman, & Ahissar, 2005). These back-and-forth sweeps or cycles result in the whiskers bending when they come into contact with an object or surface, which exerts forces on the follicle sinus at the base of each whisker (Sofroniew et al., 2014). A pathway of three synapses links the primary afferents (from the whisker follicle receptors on one side of the face to the contralateral cortex) to the final link made into Layer IV, producing the barrel pattern identified in cross-section through this layer (Van der Loos & Woolsey, 1972). The topological positions of the barrels match the positions of whiskers on the face, with each whisker corresponding to a single barrel. A recent study used two-photon calcium imaging to assess the responses of Layer II and III neurons in S1 to stimulating isolated whiskers with sandpapers of differing coarsenesses (P120, P320, P600, and P1000; Garion et al., 2014). This study demonstrated that many neurons have a preferred coarseness, with a minority showing monotonic increases in response to increases or decreases in the degree of coarseness. Also, neurons from columns that were close together tended prefer the same coarseness, and those at different depths within a column responded to the same coarseness.

The barrel cortex changes in response to altered tactile experience in both young and adult rodents. For example, Wallace and Fox (1999) trimmed the whiskers of adolescent rats in a chessboard pattern, depriving every other whisker. After seven days of deprivation, the capacity of the spared whiskers and regrown whiskers to provoke activity in their associated barrels was assessed, revealing an increased response to the spared whisker and a reduced response to the trimmed whisker (see also Diamond, Armstrong-James, & Ebner, 1993; Shepherd, Pologruto, & Svoboda, 2003; Simons & Land, 1994). These deprivation experiments also reveal an expansion in the area of cortex activated by spared whiskers, which is mirrored by a reduction in the area activated by the deprived whisker. Importantly, expansion of the spared whisker domains arises from synaptic plasticity in the somatosensory cortex and can be prevented by blocking cortical activity during acquisition (Wallace, Glazewski, Liming, & Fox, 2001) or by inactivating the spared whisker’s barrel in Layer IV during expression of the plasticity (Fox, 1994; Fox, Wright, Wallace, & Glazewski, 2003). Plasticity therefore takes place in the same cortical area where texture learning is coded. How might such plasticity be related to perceptual learning, which relies on a reduction in the processing of the elements that the to-be-discriminated stimuli share?

One possibility follows from the idea that when a rodent is presented with a novel tactile stimulus (e.g., C) in a specific context (an arena), whisking results in a specific pattern of activity across the barrel cortex that becomes represented as functional changes within the barrel map. Now suppose that this pattern of activation can be produced either when the animal encounters the same stimulus again or when the animal is placed in the context in which the stimulus was originally encountered. If the processing of information from the whiskers is reduced when there is a match between stimulus-driven activation of the barrel cortex and the associatively retrieved activation, this could provide a basis both for stimulus-specific habituation (see Wagner, 1981) and for perceptual learning that relies on such long-term habituation (Montuori & Honey, 2016). For example, recent activation of the barrel cortex that is a result of either recent direct activation (cf. Wu et al., 2013) or associative activation could result in a refractory process that reduces the impact of whisker-generated information. This type of explanation for changes in stimulus processing has been applied successfully in a variety of behavioral systems, including control of the orienting response in rats (Honey & Good, 2000) and object recognition (Robinson & Bonardi, 2015; Sanderson, 2016), as well as those systems that formed the basis of the original idea (Wagner, 1981).

## Concluding comments

Theoretical models of associative learning assume a restricted set of general processes with broad applicability. Rodents have been the favored experimental mammal in studies of the nature of associative processes, but behavioral investigation of learning and memory in rats and mice has tended to use procedures that have not allowed ready consideration of the sensory systems involved. Here we have focused on perceptual learning involving tactile stimuli in rodents, in which preliminary behavioral analysis can be brought together with the literature on the neural basis of somatosensory processing and plasticity. We have offered some speculation about how perceptual learning (and habituation) might be engendered through the interaction between active touch—involving the whiskers—and the barrel cortex. Further research will be necessary to determine whether or not this analysis is accurate.
